# Trends of serotypes and resistance among *Streptococcus pneumoniae* in the UK and Ireland (1999–2019)

**DOI:** 10.1093/jac/dkaf253

**Published:** 2025-10-27

**Authors:** Carolyne Horner, Rosy Reynolds, Shazad Mushtaq, Aiysha Chaudhry, Rachael Adkin, Michael Allen, Christopher Longshaw, Benjamin J Parcell, David M Livermore

**Affiliations:** British Society for Antimicrobial Chemotherapy, 53 Regent Place, Birmingham B1 3NJ, UK; British Society for Antimicrobial Chemotherapy, 53 Regent Place, Birmingham B1 3NJ, UK; Population Health Sciences, University of Bristol, Bristol BS8 2PS, UK; Antimicrobial Resistance and Healthcare Associated Infections Reference Unit, UK Health Security Agency, Colindale, London NW9 5EQ, UK; Antimicrobial Resistance and Healthcare Associated Infections Reference Unit, UK Health Security Agency, Colindale, London NW9 5EQ, UK; Antimicrobial Resistance and Healthcare Associated Infections Reference Unit, UK Health Security Agency, Colindale, London NW9 5EQ, UK; British Society for Antimicrobial Chemotherapy, 53 Regent Place, Birmingham B1 3NJ, UK; Medical Affairs, MSD (UK) Limited, 120 Moorgate, London EC2M 6UR, UK; British Society for Antimicrobial Chemotherapy, 53 Regent Place, Birmingham B1 3NJ, UK; Scientific Affairs, Shionogi B.V., Fifty Paddington, 50 Eastbourne Terrace, Paddington W2 6LG, UK; Division of Population Health and Genomics, School of Medicine, University of Dundee, Ninewells Hospital and Medical School, Dundee DD1 9SY, UK; Department of Medical Microbiology, Ninewells Hospital and Medical School, Dundee DD1 9SY, UK; Antimicrobial Resistance and Healthcare Associated Infections Reference Unit, UK Health Security Agency, Colindale, London NW9 5EQ, UK; Norwich Medical School, University of East Anglia, Norwich NR4 7TJ, UK

## Abstract

**Objectives:**

This study aimed to report the serotype distribution of *Streptococcus pneumoniae* isolates from UK and Irish patients with bacteraemia or community-associated lower respiratory tract infections (CA-LRTI). Depending upon the year, these were from 23 to 39 sentinel laboratories and were collected between 1999 and 2019, thus spanning the introduction of pneumococcal conjugate vaccines, PCV7 and PCV13.

**Methods:**

Pneumococcal identification, susceptibility testing and serotyping were undertaken by a central laboratory. Changes in serotype distributions and among the predominant types showing antibiotic non-susceptibility were reviewed in relation to vaccine deployment.

**Results:**

Following the introduction of PCV7 (2006) and PCV13 (2010), major shifts occurred in serotype prevalence for both bacteraemia and CA-LRTI. PCV7 types and most PCV13 types (but not 3 and 19A) were largely or wholly displaced. Many of the displaced types (e.g. 6B, 9V, 14, 19F and 23B) had been internationally prevalent and were associated with antibiotic resistance. Other serotypes—many included within the older pneumococcal polysaccharide vaccine, PPV23—expanded into the space, with serotype 8 becoming especially prominent in bacteraemia, though not respiratory infections. Further increasingly prevalent types included 9N, 10A, 12F and 22F. Serotype 15A, often multi-resistant, rose then fell in relative importance after deployment of PCV13. Among the currently most prevalent types, serotype 3 is rarely resistant to agents besides tetracyclines and bloodstream serotype 8 isolates mostly are fully susceptible.

**Conclusions:**

These data offer a comparison of serotypes associated with bacteraemia and respiratory disease over two decades in the UK and Ireland.

## Introduction

*Streptococcus pneumoniae* infections remain major causes of morbidity and mortality, especially among children aged <5 years, adults aged ≥65 years and those with compromised immunity or other underlying medical conditions.^1,2^ A major virulence factor of the species is its polysaccharide capsule, which impedes phagocytosis. Capsular polysaccharide antigens are used to classify *S. pneumoniae* into >100 serotypes.^3^ These differ in their capability to cause disease, with 30 serotypes accounting for over 90% of cases.^4^

Vaccines have been developed to protect at-risk individuals and to reduce the incidence of invasive pneumococcal disease (IPD). In the UK, an inactivated 23-valent pneumococcal polysaccharide vaccine (PPV23) has been recommended since 1992 for at-risk individuals aged ≥2 years and was introduced in three stages for older individuals: in August 2003 for people aged ≥80 years, April 2004 for those of ≥75 years and April 2005 for those of ≥65 years.^5^ However, PPV23 lacks efficacy in children < 2 years, and conjugate vaccines (PCVs) were developed primarily for this age group. These have reduced the incidence of IPD in both the vaccinated children and, by a herd effect, in adults. PCV7 (Prevenar7^®^, Wyeth, later Pfizer) was added to the UK immunization programme for infants < 2 years old in September 2006, with a catch-up programme for children born from September 2004 onwards. PCV13 (Prevenar13^®^, Pfizer) replaced PCV7 in the childhood immunization schedule in April 2010, covering more serotypes, including some (e.g. 19A) that had proliferated since deployment of PCV7.^2^ While PCVs have reduced the incidence of IPD,^6–8^ concern remains that they are being undermined by the replacement of vaccine types by non-vaccine types.^9–11^ This is leading to the ongoing development of broader spectrum PCVs, likely to be adopted in the future. The coverage of current and developmental vaccines is summarized in Table 1.

**Table 1.** Pneumococcal vaccines (UK)

Vaccine type	Licensed name (manufacturer)	Serotypes coverage	Additional information
Pneumococcal polysaccharide vaccine (PPV23)	Pneumovax^®^ 23 (MSD)	1, 2, 3, 4, 5, 6B, 7F, 8, 9N, 9V, 10A, 11A, 12F, 14, 15B, 17F, 18C, 19A, 19F, 20, 22F, 23F, 33F	Recommended for clinical risk groups since 1992. Immunization for older adults was introduced in three stages: in August 2003 for people aged ≥80 years, April 2004 for those of ≥75 years and April 2005 for those of ≥65 years
Pneumococcal conjugate vaccine (PCV7)	Prevenar7^®^ (Pfizer, previously Wyeth)	4, 6B, 9V, 14, 18C, 19F, 23F	Introduced into the routine childhood immunization programme in the UK in September 2006 with a catch-up programme to cover children born from September 2004 onwards. It was replaced by PCV13 in April 2010
Pneumococcal conjugate vaccine (PCV10)	Synflorix^®^ (GSK)	1, 4, 5, 6B, 7F, 9V, 14, 18C, 19F, 23F.	Approved for use by the European Medicines Agency in March 2009 and licensed for use in the UK. Not included in the UK national immunization programme
Pneumococcal conjugate vaccine (PCV13)	Prevenar13^®^ (Pfizer)	1, 3, 4, 5, 6A, 6B, 7F, 9V, 14, 18C, 19A, 19F, 23F	PCV13 replaced PCV7 in April 2010. JCVI gives the options that both PCV13 and PCV15 are acceptable for use in the infant programme. At time of writing UKHSA lists Prevenar 13 as the vaccine to use as part of routine childhood immunizations
Pneumococcal conjugate vaccine (PCV15)	Vaxneuvance^®^ (MSD)	1, 3, 4, 5, 6A, 6B, 7F, 9V, 14, 18C, 19A, 19F, 22F, 23F, 33F	PCV15 was licensed for use in 2022, from 6 weeks of age and has broader strain coverage, but there is less certainty about the protection derived from a 1 + 1 schedule. The Green Book^a^ and the JCVI^b^ advise that both PCV13 and PCV15 can be used in the infant vaccination programme; however, providers should offer the vaccine supplied for the infant programme at that time
Pneumococcal conjugate vaccine (PCV20)	Prevenar 20^®^ (Pfizer)	1, 3, 4, 5, 6A, 6B, 7F, 8, 9V, 10A, 11A, 12F, 14, 15B, 18C, 19A, 19F, 22F, 23F, 33F	Licensed for use in both paediatrics and adults. Not currently included in the UK national immunization programme
Pneumococcal conjugate vaccine (PCV21)	V116 (MSD)	3, 6A, 7F, 8, 9N, 10A, 11A, 12F, 15A, 15C, 16F, 17F, 19A, 20A, 22F, 23A, 23B, 24F, 31, 33F, 35B	An investigational, 21-valent pneumococcal conjugate vaccine designed for use in adults

^a^The Green Book: immunization against infectious disease: https://www.gov.uk/government/collections/immunisation-against-infectious-disease-the-green-book#the-green-book.

^b^JCVI: Joint Committee on Vaccination and Immunization.

Much less is known or written about serotype trends in non-bacteraemia pneumonia, even though disease incidence is far higher, and the infection can be life-threatening. Likewise, there is limited information on serotype replacement in nasopharyngeal colonization in children, even though this is frequent and is a common source of transmission to older (>65 years) family members, sometimes leading to infection.

The BSAC Resistance Surveillance Project collected pneumococci from cases of community-associated lower respiratory tract infections (CA-LRTI) between 1999 and 2019 and from bacteraemia from 2001 to 2019. Here, we analyse and report the serotype distributions of these isolates, with reference to vaccine coverage. The antimicrobial susceptibility of these isolates is described elsewhere in this Supplement.^12^

## Materials and methods

The BSAC Resistance Surveillance Project was fully described previously.^13^ A detailed description of isolate collection strategies, methods of identification, susceptibility testing and serotyping is provided elsewhere in this Supplement.^14^ Briefly, fixed annual quotas of pneumococci from bacteraemia and CA-LRTIs were requested from participating microbiology laboratories in the UK and Ireland (Table S1, available as Supplementary data at *JAC* Online). Numbers of laboratories varied according to year (range 23–39 sites) (Table S2). Collection of bloodstream isolates (approx. 250 annually) was by calendar year. Pneumococci from CA-LRTIs (i.e. from community patients or those hospitalized for ≤48 h) were collected by ‘respiratory season’ to avoid splitting winter peaks between years. The first eight seasons (1999/2000–2007/08) ran from 1 October to 30 April; later collections (2008/09–2018/19) ran from 1 October to 30 September (Table S1). Submitted isolates were confirmed as pneumococci by colony appearance on horse blood agar and optochin susceptibility. Serotypes of bloodstream isolates were determined throughout the Surveillance, from 2001 to 2019; serotypes of CA-LRTI isolates were determined in the 2005/06 season and then for all seasons from 2013/14 onwards. Serotypes were determined using classical methods in most years but were inferred from WGS in 2019. MICs were determined by BSAC agar dilution.

Here, we focus on three antimicrobials used to treat *S. pneumoniae* infections that were tested in all years—penicillin, erythromycin and tetracycline; fuller susceptibility data are presented elsewhere in this Supplement.^12,15^ Isolates were categorized as S (susceptible), I (‘susceptible, increased exposure’) or R (resistant) by EUCAST breakpoints (v12.0, 2022).^16^ We refer to isolates with penicillin MIC > 0.06 mg/L and resistance to both erythromycin and tetracycline as ‘triple-resistant’.

### Vaccine groups and serotype diversity

Isolates were assigned to three overlapping vaccine groups: (i) PCV7, comprising serotypes 4, 6B, 9V, 14, 18C, 19F and 23F; (ii) PCV13, comprising PCV7 coverage plus serotypes 1, 3, 5, 6A, 7F and 19A, with the latter six serotypes referred to as ‘PCV13-non7 serotypes’; and (iii) PPV23, comprising PCV13 coverage, except 6A, plus 11 ‘PPV23-nonPCV’ serotypes: 2, 8, 9N, 10A, 11A, 12F, 15B, 17F, 20, 22F and 33F. Serotypes not represented in any of these three vaccines were categorized as ‘non-vaccine types’, as were isolates that failed to agglutinate with any antiserum. We interpreted vaccine groups strictly, assigning only isolates with the specific antigenic determinants and excluding those of the same serogroup that were identified less precisely (e.g. isolates identified only as serotype 15 or 15B/C were not assigned, whereas isolates identified as 15B were assigned).

### Statistical analysis

Analysis was descriptive and largely graphical, using Stata 18.0 (StataCorp LLC: College Station, TX) and Bischoff's colour vision-sensitive ‘plotplainblind’ graph scheme. Missing data were excluded in the calculation of percentages. Serotype diversity, excluding untyped isolates, was described using the Gini–Simpson diversity index (1 minus sum of squared probabilities).^14^

## Results

**Figure 1.**
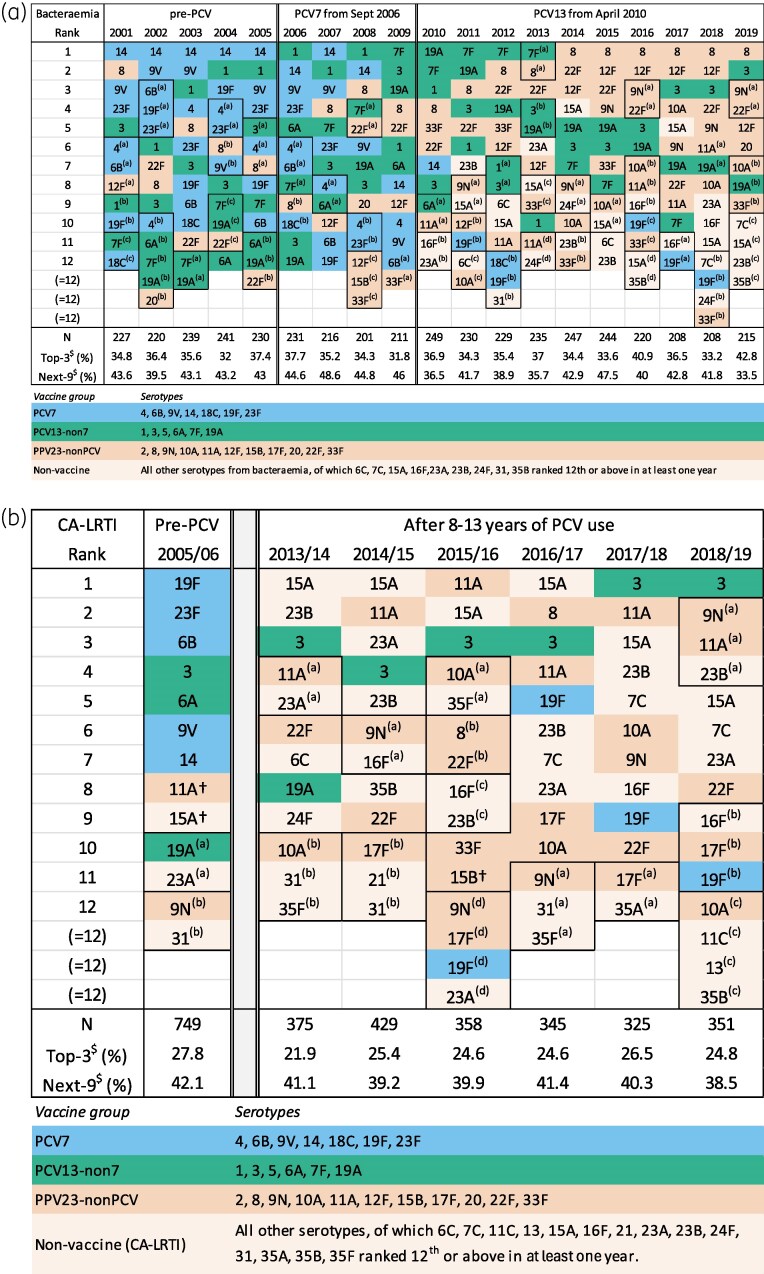
Ranked serotype distributions among isolates from (a) bacteraemia and (b) CA-LRTI indicating the top 12 serotypes by surveillance year/season. Serotypes are listed from the most prevalent to the 12^th^ (or joint 12^th^) most prevalent among *S. pneumoniae* isolates collected in each year shown. ‘Untyped’ isolates (i.e. tested, but not allocated to any serogroup) are not ranked. (Treated as a group, in CA-LRTI they would have ranked 10^th^ in 2005/06, but no higher than joint 20^th^ in any other year, and in bacteraemia, never higher than joint 15^th^.) ^(a)–(d)^Superscript letters and outlined boxes indicate serotypes with tied ranks: e.g. 4/6B/12F tied at rank 7 in bacteraemia 2001. ^†^Estimated ranks. LRTI serogroups 11 and 15 were identified only at group level in 2005/06, ranking joint eighth; ranks for 11A and 15A were based on their proportions (92% and 69%, respectively) within their serogroups in other years. Serotype ‘15B/C’ was recorded at joint eighth rank in CA-LRTI in 2015/16; among these, 15B was ranked 11^th^ based on its proportion (83%) among 15B + 15C in years when they were identified separately. In bacteraemia, 15B/C together would rank joint 12^th^ in 2015 and 2016 but neither 15B nor 15C is shown as it is unlikely that either would have done so alone. ^$^Top-3 and Next-9 record the percentage of isolates represented by the serotypes in table rows 1–3 and 4–12, respectively.

**Figure 2.**
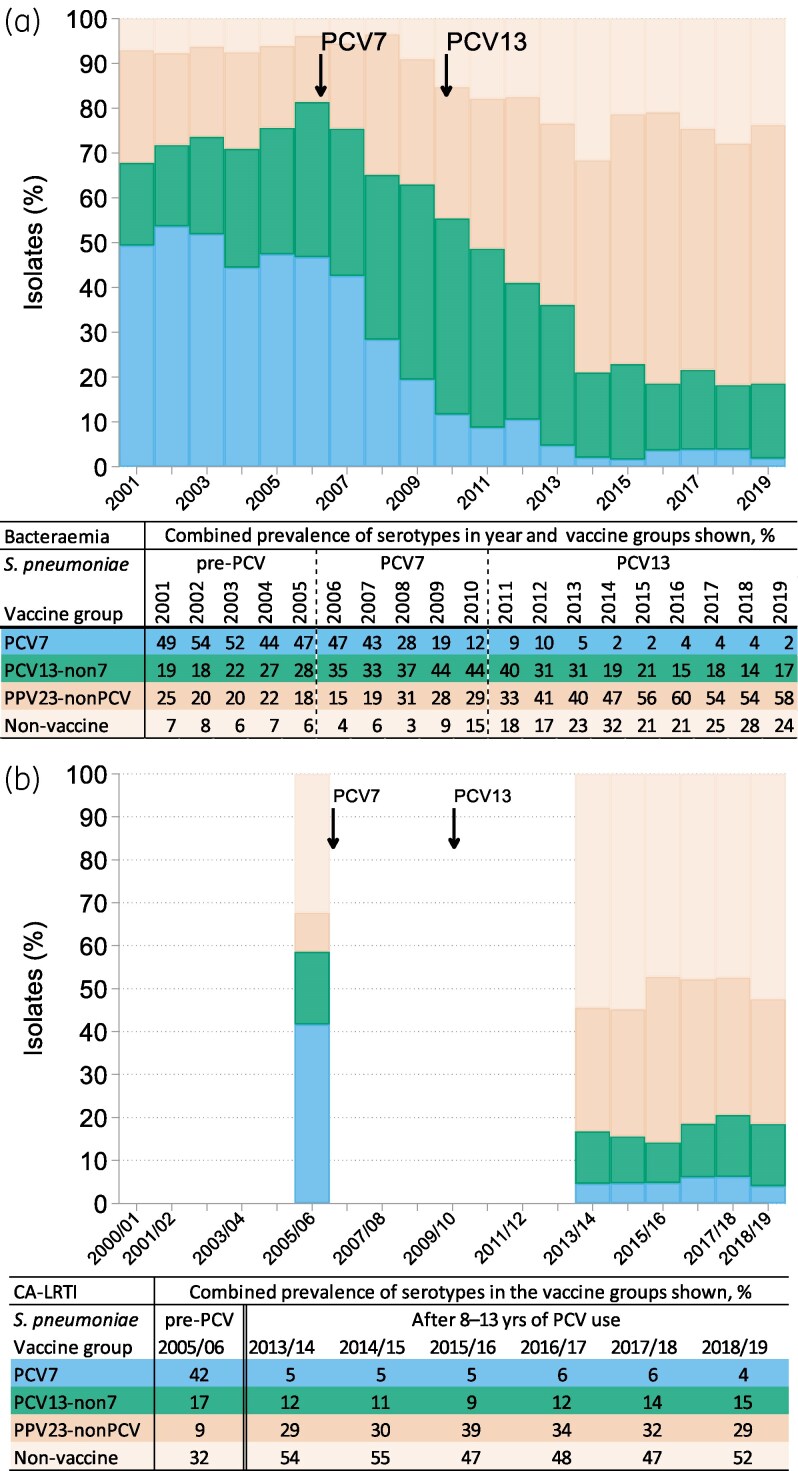
Cumulative percentage of serotypes among isolates from (a) bacteraemia and (b) CA-LRTI by vaccine group, over time. Black arrows indicate approximate introduction of PCV7 and PCV13 in England. The serotyping methods were able to identify all serogroups in these vaccines so isolates remaining untyped were treated as non-vaccine types. A few isolates with imprecise serotype identifications that prevented assignment to a vaccine type were also included as non-vaccine types. In bacteraemia, imprecise identification affected PPV23-nonPCV in 2010 and 2014–19 as some isolates of serotype 15B were recorded as 15 or 15 B/C. In CA-LRTI, in 2005/06 only, PCV13-non7 was affected by recording of 7F as 7FABC; PPV23-nonPCV was affected until 2016/17 (but mostly in 2005/06) by some recording of 11A, 15B and 33F as 11, 15, 15B/C and 33. See Figure 1 for *N* of isolates tested in each season.

**Figure 3.**
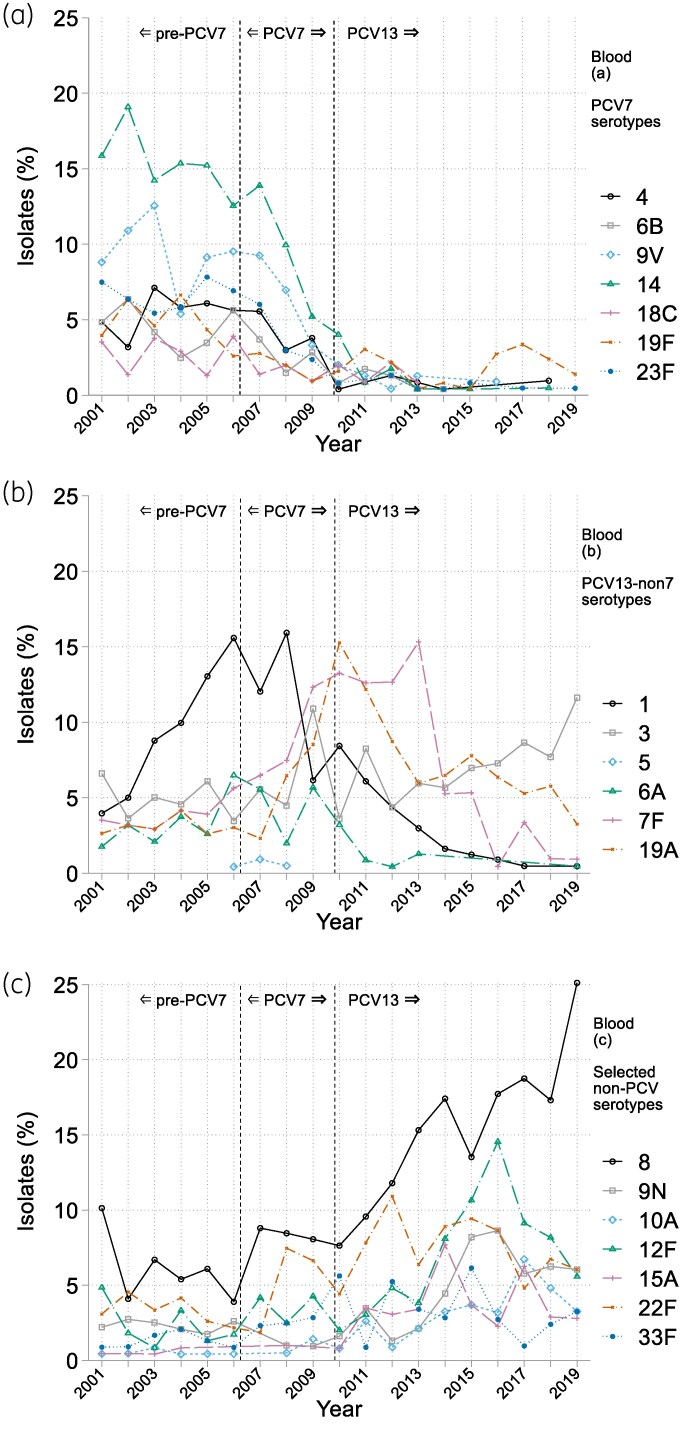
Prevalence over time of individual serotypes among bacteraemia isolates by vaccine group for (a) the seven serotypes of PCV7, (b) the additional six serotypes covered by PCV13 and (c) seven non-PCV serotypes selected as being in the top five by frequency in any surveillance year. See Figure 1 for *N* of isolates tested in each season.

**Figure 4.**
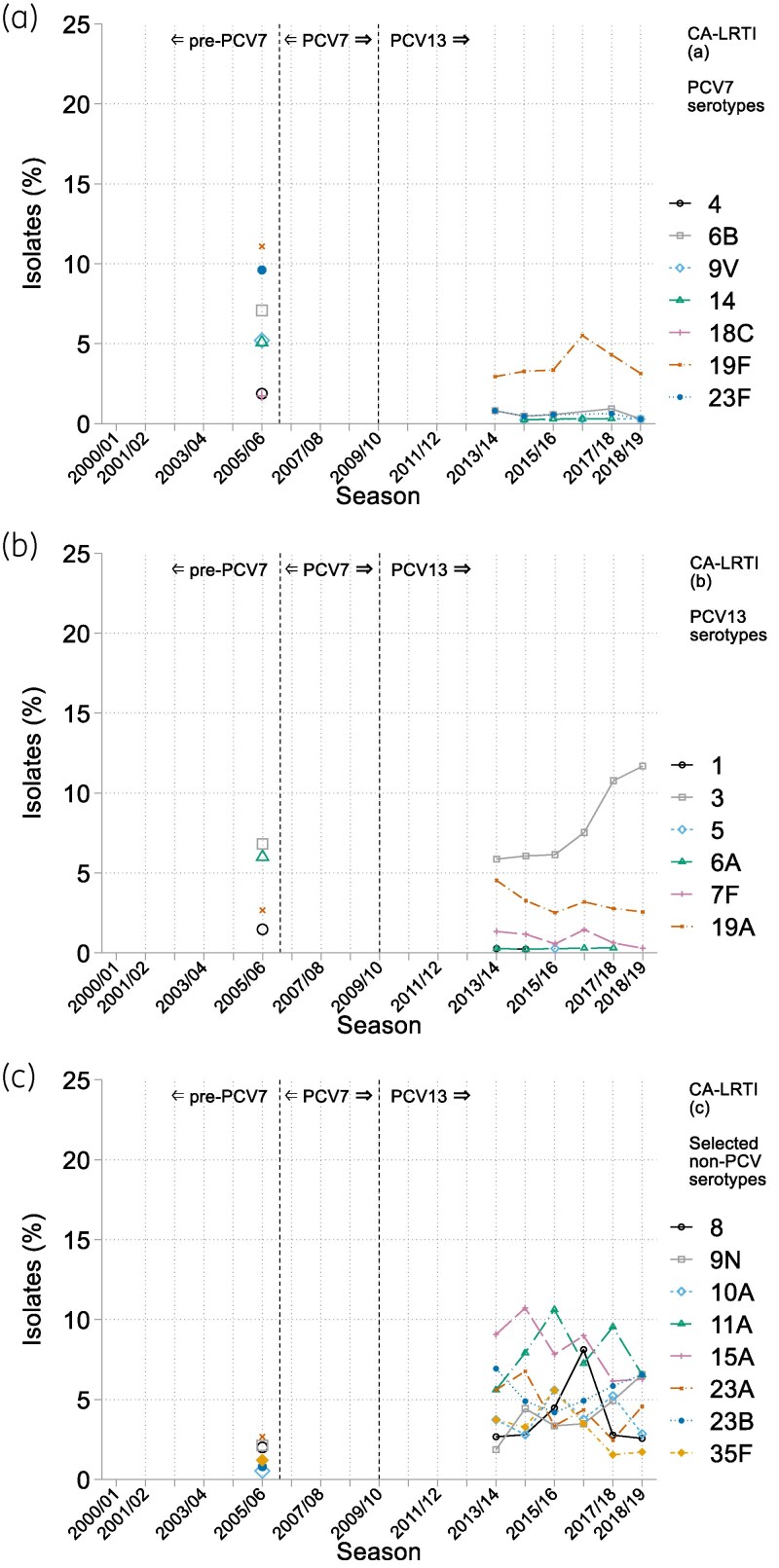
Prevalence over time of individual serotypes among CA-LRTI pneumococcal isolates by vaccine group for (a) the seven serotypes of PCV7, (b) the additional six serotypes covered by PCV13 and (c) eight non-PCV serotypes selected as being in the top four by frequency in any surveillance season. See Figure 1 for *N* of isolates tested in each season.

The BSAC Resistance Surveillance Project provides a continuous 19-year surveillance of pneumococcal serotypes in bacteraemia; isolates were grouped into descriptive periods of 5 years (2001–05) as pre-PCV and 6 years (2014–19), to represent post-PCV. For CA-LRTI isolates, the Project provides a snapshot of serotype distributions in 2005/06, the only pre-PCV season when typing was undertaken and the winter preceding PCV7 deployment, with the six respiratory seasons, 2013/14–2018/19, all of them after PCV13's introduction. The top 12 serotypes per year or season are shown in Figure 1(a and b) (bacteraemia and CA-LRTI, respectively), while serotype prevalence data over time are presented, by vaccine group, in Figure 2(a and b) for bacteraemia and CA-LRTI, respectively. Trends in the prevalence of PCV7 and 13 serotypes, along with those for selected prevalent non-PCV serotypes, are show in Figure 3(a–c) (bacteraemia) and Figure 4(a–c) (CA-LRTI). Antimicrobial resistance in relation to serotype among bacteraemia isolates is compared before and after PCV introduction in Table 2 (prevalence of resistance by serotype) and Table 3 (most prevalent serotypes among resistant isolates). Tables 4 and 5 present equivalent data for isolates from CA-LRTI. In Table 6, serotype distributions in children versus adults before and after PCV deployment are compared. Finally, Table 7 summarizes the prevalence of PPV23-non-PCV13 serotypes before and after PCV introduction by age group (0–64 years and >65 years).

**Table 2.** Non-susceptibility to penicillin, erythromycin and tetracycline before and after PCV introduction in selected serotypes of pneumococcal isolates from bacteraemia

Blood		Before PCV introduction: 2001–05					After 8–13 years of PCV use: 2014–19				
		*N* = 1157					*N* = 1342				
	Vaccine	Isolates	PEN^b^	ERY^c^	TET^d^	Triple^d^	Isolates	PEN^b^	ERY^c^	TET^d^	Triple^d^
Serotype	group^a^	*N* (%)	% I + R	%R	%R	%R	N (%)	% I + R	%R	%R	%R
1	13-non7	95 (8.2%)	0	3	2	0	11 (0.8%)^g^	x	x	x	x
3	13-non7	60 (5.2%)	0	5	3	0	106 (7.9%)	0	0	21	0
4	7	63 (5.4%)	2	2	2	0	3 (0.2%)^g^	x	x	x	x
6A	13-non7	31 (2.7%)^f^	3^f^	13^f^	0^f^	0^f^	1 (0.1%)^g^	x	x	x	x
6B	7	49 (4.2%)^f^	10^f^	16^f^	16^f^	8^f^	1 (0.1%)^g^	x	x	x	x
7F	13-non7	41 (3.5%)^f^	0^f^	0^f^	0^f^	0^f^	38 (2.8%)	0^f^	0^f^	0^f^	0^f^
8	23-nonPCV	75 (6.5%)	1	7	4	0	244 (18.2%)	0	0	1	0
9N	23-nonPCV	26 (2.2%)	0^f^	8^f^	0^f^	0	88 (6.6%)	1	0	0	0
9V	7	108 (9.3%)	37	11	4	1	2 (0.1%)	x	x	x	x
10A	23-nonPCV	6 (0.5%)^g^	x	x	x	x	55 (4.1%)	5	2	2	2
12F	23-nonPCV	28 (2.4%)^f^	0^f^	7^f^	4^f^	0^f^	126 (9.4%)	2	6	10	0
14	7	184 (15.9%)	7	58	1	1	2 (0.1%)^g^	x	x	x	x
15A	none	5 (0.4%)^g^	x	x	x	x	58 (4.3%)	33	36	28	21
18C	7	30 (2.6%)^f^	7^f^	3^f^	3^f^	0^f^	0 (0%)^g^	x	x	x	x
19A	13-non7	36 (3.1%)^f^	3^f^	8^f^	11^f^	3^f^	79 (5.9%)	15	11	10	10
19F	7	60 (5.2%)	3	5	3	2	24 (1.8%)^f^	17^f^	29^f^	25^f^	8^f^
22F	23-nonPCV	41 (3.5%)^f^	0^f^	0^f^	0^f^	0^f^	101 (7.5%)	0	0	0	0
23F	7	76 (6.6%)	7	12	7	5	5 (0.4%)^g^	x	x	x	x
33F	23-nonPCV	16 (1.4%)^g^	x	x	x	0	42 (3.1%)^f^	0^f^	33^f^	24^f^	0
ALL^e^	N/A	1157 (100%)	6.5	14.9	3.2	1.0	1342 (100%)	6.2	5.9	7.4	2.6

Clindamycin resistance was 1.8% in 2001–05 and 4.6% in 2014–19 but is not tabulated as not relevant to treatment or to definition of triple resistance. Inducible clindamycin resistance was rare at 0.1% (2/1796, tested only in 2012–19).

^a^Vaccine group: 7, in PCV7; 13-non7, in PCV13 but not PCV7; 23-nonPCV, in PPV23 but not PCV7/13; none, not included in PCV7/13 or PPV23. The non-PCV serotypes included are those that were in top five by frequency in any year. Serotype 5 (PCV13) is not shown as it was not identified in the bacteraemia isolate collection in these years.

^b^PEN: penicillin I + R, MIC > 0.06 mg/L.

^c^ERY: erythromycin R, MIC > 0.5 mg/L.

^d^TET: tetracycline R, MIC > 2mg/L; Triple: PEN I + R *and* ERY-R *and* TET-R.

^e^All serotypes in collection in these periods (48 types in 2001–05; 53 in 2014–19).

^f^Caution: 25–50 isolates, very imprecise estimation of percentages.

^g^Caution: Fewer than 25 isolates, percentages not shown.

**Table 3.** Most prevalent pneumococcal serotypes among resistant isolates from bacteraemia

Blood	(Percentage of all resistance to the antibiotic accounted for by members of the most-frequently-resistant serotypes) [cumulative percentage]							
Period	Pre-PCV7: 2001–05				After 8–13 years of PCV use: 2014–19			
Rank	Erythromycin R	Penicillin I + R	Tetracycline R	Triple R	Erythromycin R	Penicillin I + R	Tetracycline R	Triple R
	*N* = 172	*N* = 75^†^	*N* = 37^††^	*N* = 12^††^	*N* = 79^†^	*N* = 83^†^	*N* = 99^†^	*N* = 35^††^
1	14 (62) [62]	9V(53) [53]	6B(22) [22]	6B ^(=1)^ (33) [33]	15A(27)[27]	15A(23)[23]	3(22)[22]	15A(34)[34]
2	9V(7)[69]	14(16)[69]	23F(14)[35]	23F ^(=1)^ (33) [67]	33F(18)[44]	23B(20)[43]	15A(16)[38]	19A(23)[57]
3	23F(5)[74]	6B^(=3)^(7)[76]	9V^(=3)^(11)[46]	9V^(=3)^(8)[75]	19A(11)[56]	19A(14)[58]	12F(12)[51]	23F(9)[66]
4	6B(5)[79]	23F^(=3)^(7)[83]	19A^(=3)^(11)[57]	14^(=3)^(8)[83]	12F(10)[66]	19F^(=4)^(5)[63]	33F(10)[61]	19F^(=4)^(6)[71]
5	8(3)[82]	18C^(=5)^(3)[85]	8(8)[65]	19A^(=3)^(8)[92]	19F(9)[75]	35B^(=4)^(5)[67]	19A(8)[69]	38^(=4)^(6)[77]
6	6A(2)[84]	19F^(=5)^(3)[88]	1^(=6)^(5)[70]	19F^(=3)^(8)[100]	11A^(=6)^(4)[78]	10A^(=6)^(4)[71]	19F(6)[75]	8 types equal^c^
7	1^(=7)^(2)[86]	35B^(=5)^(3)[91]	3^(=6)^(5)[76]		23F^(=6)^(4)[82]	12F^(=6)^(4)[75]	23F(3)[78]	
8	3^(=7)^(2)[88]	7 types^a^[100]	19F^(=6)^(5)[81]		6C^(=8)^(3)[85]	23F^(=6)^(4)[78]	6 types equal^b^	
9	19A^(=7)^(2)[90]				9V^(=8)^(3)[87]			
10	19F^(=7)^(2)[91]				38^(=8)^(3)[90]			

Serotypes are listed down to the eighth (or joint eighth) most frequent among isolates with the specified resistance in the period shown. Untyped isolates are included in calculation of prevalence but, even treated as a single group, did not attain eighth or higher rank as contributors of these resistances in bacteraemia.

^a^Seven serotypes of equal eighth rank, one isolate (1.3%) each of 4, 6A, 8, 15A, 19A, 22A, 23A.

^b^Six serotypes of equal eighth rank, two isolates (2.0%) each of 6C, 8, 11A, 16F, 35F, 38.

^c^Eight serotypes of equal sixth rank, one isolate (2.9%) each of 6B, 6C, 7C, 9V, 10A, 11A, 15C, 24F.

^†^Caution: note small^†^ (<100) or very small^††^ (<50) numbers for most resistances in both periods.

^(=n)^ = equal n^th^ rank, listed in order by serogroup, then serotype.

**Table 4.** Non-susceptibility to penicillin, erythromycin and tetracycline before and after PCV introduction in selected serotypes of pneumococcal isolates from CA-LRTI

CA-LRTI		Before PCV introduction: 2005/06					After 8–13 years of PCV use: 2013/14–2018/19				
		*N* = 749					*N* = 2183				
	Vaccine group^a^	Isolates	PEN^b^	ERY^c^	TET^d^	Triple^e^	Isolates	PEN^b^	ERY^c^	TET^d^	Triple^e^
Serotype		*N* (%)	% I + R	%R	%R	%R	*N* (%)	% I + R	%R	%R	%R
1	13-non7	11 (1.5%)^x^	x	x	x	x	2 (0.1%)^x^	x	x	x	x
3	13-non7	51 (6.8%)	0	0	0	0	172 (7.9%)	2	4	27	1
4	7	14 (1.9%)^x^	x	x	x	x	0 (0%)^x^	x	x	x	x
5	13-non7	0 (0%)^x^	x	x	x	x	1 (0%)^x^	x	x	x	x
6A	13-non7	45 (6.0%)^h^	4^h^	4^h^	4^h^	0^h^	4 (0.2%)^x^	x	x	x	x
6B	7	53 (7.1%)	9	21	21	9	11 (0.5%)^x^	x	x	x	x
7F	13-non7	0 (0%)^x^	x	x	x	x	20 (0.9%)^x^	x	x	x	x
8	23-nonPCV	15 (2.0%)^x^	x	x	x	x	84 (3.8%)	4	6	10	4
9N	23-nonPCV	16 (2.1%)^x^	x	x	x	x	89 (4.1%)	3	3	4	0
9V	7	39 (5.2%)^h^	31^h^	13^h^	5^h^	5^h^	3 (0.1%)^x^	x	x	x	x
10A	23-nonPCV	4 (0.5%)^x^	x	x	x	0	86 (3.9%)	2	5	3	2
11A/11^g^	23-nonPCV	36 (4.8%)^h,g^	0^h,g^	0^h,g^	0^h,g^	0^h,g^	172 (7.9%)	5	7	3	2
14	7	38 (5.1%)	34^h^	61^h^	24^h^	21^h^	4 (0.2%)^x^	x	x	x	x
15A/15^g^	None	36 (4.8%)^h,g^	0^h,g^	17^h,g^	11^h,g^	0^h,g^	181 (8.3%)	41	52	46	35
18C	7	13 (1.7%)^x^	x	x	x	x	0 (0%)^x^	x	x	x	x
19A	13-non7	20 (2.7%)^x^	x	x	x	x	69 (3.2%)	41	39	38	32
19F	7	83 (11.1%)	10	24	16	5	81 (3.7%)	38	62	59	33
23A	None	20 (2.7%)^x^	x	x	x	x	101 (4.6%)	9	8	7	4
23B	None	6 (0.8%)^x^	x	x	x	x	121 (5.5%)	29	10	2	2
23F	7	72 (9.6%)	6	4	7	3	10 (0.5%)^x^	x	x	x	x
35F	None	9 (1.2%)^x^	x	x	x	x	71 (3.3%)	3	7	6	0
All^f^	N/A	749 (100%)	6.8	11.6	8.4	3.1	2183 (100%)	13.7	17.4	15.3	7.3

Clindamycin resistance was 5.1% in 2005/06 and 13.2% in 2013/14–18/19 but is not tabulated as not relevant to treatment or to definition of triple resistance. Inducible clindamycin resistance was rare at 0.4% (11/2911, tested only in 2011/12–2018/19).

^a^Vaccine group: 7, in PCV7; 13-non7, in PCV13 but not PCV7; 23-nonPCV, in PPV23 but not PCV7/13; none, not included in PCV7/13 or PPV23. The non-PCV serotypes included are those that were in top four by frequency in any surveillance season (October–April in 2005/06; October–September in 2013/14–2018/19).

^b^PEN: penicillin I + R, MIC > 0.06 mg/L.

^c^ERY: erythromycin R, MIC > 0.5 mg/L.

^d^TET: tetracycline R, MIC > 2mg/L.

^e^Triple: PEN I + R *and* ERY-R *and* TET-R.

^f^All serotypes in collection in these periods (49 types in 2005/06; 74 in 2013/14–2018/19).

^g^Counts and estimates are for serogroups 11 and 15 in 2005/06 (pre-PCV) and for serotypes 11A and 15A in 2013/14–2018/19 (post-PCV). (In the later period, when they were identified individually, these two serotypes made up about 90% (11A) and 70% (15A) of their respective serogroups.)

^h^Caution: 25–50 isolates, very imprecise estimation of percentages.

^x^Caution: Fewer than 25 isolates, percentages not shown.

**Table 5.** Most prevalent pneumococcal serotypes among resistant isolates from CA-LRTI

CA-LRTI	Most prevalent serotypes in resistant isolates by period and antimicrobial resistance: serotype (%) [cumulative %]							
Period	Before PCV introduction 2005/06				After 8–13 years of PCV use 2013/14–2018/19			
Rank	Erythromycin R	Penicillin I + R	Tetracycline R	Triple R	Erythromycin R	Penicillin I + R	Tetracycline R	Triple R
	*N* = 87^†^	*N* = 51^†^	*N* = 63^†^	*N* = 23^††^	*N* = 380	*N* = 298	*N* = 335	*N* = 160
1	14(26)[26]	14(25)[25]	19F(21)[21]	14(35)[35]	15A(25)[25]	15A(25)[25]	15A(25)[25]	15A(40)[40]
2	19F(23)[49]	9V(24)[49]	6B(17)[38]	6B(22)[57]	19F(13)[38]	23B(12)[37]	19F(14)[39]	19F(17)[57]
3	6B(13)[62]	19F(16)[65]	14(14)[52]	19F(17)[74]	19A^(=3)^(7)[45]	35B(11)[48]	3(14)[53]	19A(14)[71]
4	15(7)[69]	6B(10)[75]	23F(8)[60]	9V^(=4)^(9)[83]	33F^(=3)^(7)[52]	19F(10)[58]	19A(8)[61]	6B(6)[76]
5	9V(6)[75]	23F(8)[82]	15^(a)^(6)[67]	23F^(=4)^(9)[91]	35B(6)[58]	19A(9)[67]	33F(6)[67]	23A(3)[79]
6	23F(3)[78]	6A^(=6)^(4)[86]	28A(5)[71]		6C(4)[62]	6B(3)[71]	6C(4)[70]	8^(=6)^(2)[81]
7	6A^(=7)^(2)[80]	9A^(=6)^(4)[90]	6A^(=7)^(3)[75]		11A^(=7)^(3)[65]	11A^(=7)^(3)[74]	6B(3)[73]	11A^(=6)^(2)[83]
8	33A^(=7)^(2)[83]	19A^(=8)^(2)[92]	9V^(=7)^(3)[78]		23B^(=7)^(3)[68]	23A^(=7)^(3)[77]	8(2)[75]	12F^(=6)^(2)[84]
9	34^(=7)^(2)[85]	35B^(=8)^(2)[94]	33A^(=7)^(3)[81]					
10		36^(=8)^(2)[96]						

Serotypes are listed down to the eighth (or joint eighth) most frequent among isolates with the specified resistance in the period shown. Untyped isolates are included in calculation of prevalence but not shown because not a defined serotype. Tabulated as a single type, they would rank between fourth and sixth as contributors of each of the four resistances in LRTI in 2005/06 and, by a different serotyping method, sixth and eighth for triple and tetracycline resistances in 2013/14–18/19.

^†^Caution: note small^†^ (<100) or very small^††^(<50) numbers in the single year's data before PCV introduction.

^(a)^Serogroup 15 was not identified to serotype level in LRTI in 2005/06 (pre-PCV).

^(=n)^ =equal n^th^ rank, listed in order by serogroup, then serotype.

**Table 6.** Percentage of isolates from bacteraemia and CA-LRTI by vaccine group, in pre-vaccine and recent periods, according to eligibility to receive PCV7/13

	Percentage of isolates in vaccine groups							
	Bacteraemia				Lower respiratory tract infections			
	Age ≤ 9 years^a^ (eligible)		Age ≥ 16 years^b^ (not eligible)		Age ≤ 9 years^a^ (eligible)		Age ≥ 16 years^b^ (not eligible)	
Season	2001–05	2014–19	2001–05	2014–19	2005/06	2013/14–2018/19	2005/06	2013/14–2018/19
Vaccine group	*N* = 126^d^	*N* = 80^d^	*N* = 1010	*N* = 1250	*N* = 32^d^	*N* = 78^d^	*N* = 703	*N* = 2085
PCV7	76	6	46	3	44	4	42	5
PCV13-non7	14	18	24	17	6	10	17	12
PPV23-nonPCV	5	38	23	56	3	31	9	32
Non-vaccine^c^	6	39	7	24	47	55	32	51

Percentages may not sum to 100% because of rounding errors.

PCV13-non7: serotypes in PCV13 but not PCV7; PPV23-nonPCV: serotypes in PPV23 but not PCV7 or PCV13.

^a^Age ≤ 9 years: represent a group who, for the more recent period reviewed, were likely to have received PCV7 or PCV13. PCV7 was introduced in September 2006 with a catch-up programme reaching back to children born in or after September 2004. The oldest of those born in or after September 2004 would turn 9 years old in September 2013, i.e. just before the start of the post-PCV analysis period.

^b^Age ≥ 16 years: represent a group who were not eligible to receive PCV7 or 13 in the study period. Those born before September 2004 would turn 15 years old as the BSAC Surveillance Project ended (December 2019) and would have missed vaccination in the UK childhood immunization schedule.

^c^Non-vaccine group comprising isolates that had serotypes not allocated to any of the vaccine groups shown.

^d^Caution: estimates for the vaccine-eligible children are based on small numbers.

**Table 7.** Frequency and prevalence of PPV23 serotypes among non-PCV13 serotypes of pneumococcal isolates from bacteraemia and CA-LRTI

Infection	Age group	Before PCV introduction		After 8–13 years of PCV use		Relative proportion^a^
		2001–05 (blood)		2014–19 (blood)		
		2005/06 (LRTI)		2013/14–2018/19 (LRTI)		Post-PCV/pre-PCV
	Years	Count	% [CI]	Count	% [CI]	ratio [CI]
Blood	0–64	124/162	76.5 [71.1–82.4]	374/498	75.1 [70.2–80.4]	0.98 [0.89–1.11]
	≥65	118/160	73.8 [67.5–80.6]	358/569	62.9 [57.1–69.3]	0.85 [0.75–0.97]
CA-LRTI	0–64	41/194	21.1 [16.1–27.7]	364/883	41.2 [38.1–44.6]	1.95 [1.47–2.59]
	≥65	27/116	23.3 [16.7–32.4]	330/920	35.9 [32.9–39.1]	1.54 [1.09–2.17]

[CI] 95% confidence intervals calculated using binomial GLM with log link and robust standard errors (respiratory) or robust standard errors for clustering by year (bacteraemia).

^a^ *N* of PPV23-type isolates/*N* of non-PCV13 isolates.

### Serotype distributions among S. pneumoniae from bacteraemia

Among 4301 pneumococcal isolates from bacteraemia, 4289 (99.7%) were assigned a serotype, with just 12 untyped or untypeable. Thirty-six serogroups and at least 64 serotypes were represented. In the first 5 years (2001–05) before introduction of PCV7, 31 serogroups and ≥47 serotypes were recorded; in the final 6 years (2014–19), 31 serogroups and ≥50 serotypes were identified. While greater serotype diversity might be expected in 6 years than 5, since the larger sample would be likely to include a wider selection of less prevalent serotypes, this was not seen. Moreover, no trend was identified in the annual serotype diversity index, which was estimated at 0.930 in 2001 and 0.903 in 2019, with a mean of 0.929 (range 0.923–0.935) for the five pre-PCV estimates (2001–05) and a mean of 0.922 (range 0.903–0.931) for the six most recent estimates (2014–19). In each year, before and after PCV deployment, the top three serotypes accounted for approximately one-third of the isolates while around 75% of isolates belonged to the top 12 serotypes (Figure 1a).

Before the introduction of PCV7, its serotypes dominated, collectively accounting for 44%–54% of bloodstream isolates during 2001–05 (Figure 2a). This proportion then declined markedly and, after 2014, PCV7 serotypes comprised <5% of isolates (Figure 2a). All PCV7 serotypes declined, but the decline was particularly striking for serotype 14 (Figure 3a). Across 2001–05, this had been the most prevalent single serotype, though with a slow reduction in relative prevalence; subsequently, it declined rapidly, accounting for 0.4% of all bloodstream pneumococci from 2011. By the time PCV13 was deployed in 2010, most PCV7 serotypes, except 19F, had left the top 12 (Figure 1a).

Collectively, PCV13-non7 serotypes increased in bacteraemia until 2010, when PCV13 replaced PCV7, and then gradually returned to pre-PCV prevalence (Figure 2a). This collective stability disguises substantial differences among individual serotypes. Before the introduction of PCV7, serotype 1 was considerably the most prevalent PCV13-non7 type and was increasing (Figure 3b). Despite not being included in PCV7, it then showed a rapid decline after 2008, which continued after the introduction of PCV13. The PCV13-non7 serotype 6A also ceased to be prominent after deployment of PCV13, while serotype 7F declined later, from a higher base. By contrast, two other PCV13-non7 serotypes, 3 and 19A, have remained prominent in bacteraemia even after the introduction of PCV13, with serotype 3 gaining importance and serotype 19A losing importance slowly over time (Figure 3b).

As the surveillance proceeded, PCV7 and PCV13-non7 vaccine types (except serotype 3 and to a degree 19A) were progressively and substantially replaced in bacteraemia by PPV23-nonPCV serotypes, notably 8, 9N, 12F and 22F. Along with serotype 3, these regularly and consistently ranked among the top five serotypes from 2014 onwards (Figure 1a). Several non-vaccine types also rose in prominence after 2010, most notably 15A, which featured among the top 12 serotypes in every year from 2011 onwards (Figure 1a), rising in importance until 2014, then declining (Figure 3c).

Overall, the proportion of PPV23-nonPCV serotypes increased from 25% in 2001% to 58% in 2019 (Figure 2a) while the percentage of non-vaccine serotypes, not covered by PCV13 nor PPV23, also rose, from 15% in 2010% to 24% in 2019 (Figure 2a). Unlike with the introduction of PCV7 (and to a lesser degree, PCV13), the wide deployment of PPV23 in adults ≥ 80 years in 2003 was not accompanied by obvious shifts in serotype distribution, with the same serotypes dominating from 2001 until the introduction of PCV7 in 2006 (Figure 1a).

### Serotype distribution among S. pneumoniae from CA-LRTI

Serotype data were available only for CA-LRTI pneumococci collected during 2005/06 (*N* = 749) (i.e. immediately before introduction of PCV7) and from 2013/14 onwards (*N* = 2183), around 7 years after PCV7 was introduced and 3 years after it was replaced by PCV13.

Thirty-seven serogroups and ≥79 distinct serotypes were identified in total. Isolates typed in 2005/06 comprised 33 serogroups and ≥48 serotypes; 30 (4.0%) isolates remained untyped. Those typed during the 2013/14–2018/19 period included 33 serogroups and ≥68 serotypes, with 16 (0.7%) isolates remaining unassigned. No trend was identified in the serotype diversity index: the average of values estimated for each of the six post-PCV seasons was 0.952 (range 0.950–0.955), compared with 0.946 in the one pre-PCV season.

In each year where typing was performed, the top three serotypes accounted for approximately one-quarter of the collection, versus 30%–40% in bacteraemia, and the top 12 serotypes for approximately 70%, a similar proportion to that in bacteraemia (Figure 1b). Pneumococci with serotypes belonging to the PCV7 spectrum comprised 42% of the 2005/06 collection (similar to 47% for both the 2005 and 2006 bacteraemia collections), but were rare from 2013/14 onwards, collectively accounting for 4%–6% of annual collections (Figure 2b); only 19F persistently accounted for more than 2% of isolates and retained a position in the top 12 (Figures 1b and 4a). Serotypes 23F, 19F and 6B experienced the largest decreases (Figure 4a).

Again, paralleling bacteraemia experience, the proportion of PCV13-non7 serotypes declined more modestly than PCV7 serotypes, from 17% in 2005/06 to average 12% in 2013/14–2018/19 (Figure 2b); only serotypes 3 and 6A accounted for more than 5% of isolates in 2005/06 (Figure 4b). Serotype 3 increased from 7% of isolates in 2005/06% to 12% in 2018/19, becoming the most prevalent CA-LRTI serotype in the two most recent years, while serotype 6A fell from 6% of isolates in 2005/06 to <1% in 2013/14–2018/19 (Figure 4b). Serotype 19A maintained its 2%–5% prevalence but, unlike in bacteraemia, lost its top 12 ranking (Figures 1b and 4b).

The percentage of PPV23-nonPCV serotype isolates rose markedly, from 9% in 2005/06 to 29%–39% from 2013/14 to 2018/19 (Figure 2b). Serotype 11A contributed most (8% points) to this increase with six other types all increasing by 2–4 points. Unlike in bacteraemia, serotype 8 did not become persistently prominent. The percentage of non-vaccine serotype isolates was higher in CA-LRTI than in bacteraemia throughout (32% in 2005/06, increasing to around 50% from 2013/14 to 2018/19 versus mean 7% for bacteraemias in 2001–05 increasing to around 25% in 2014–19) (Figure 2b and a). Prominent among the non-vaccine serotypes driving this rise in CA-LRTI were 15A, 23B and 7C, although, among these, only 15A and 23B reached individual prominence among serotypes as a whole (Figures 4c and 1b). As among bacteraemia isolates, the proportion of serotype 15A isolates increased and then declined (Figure 4c).

### Serotypes associated with antimicrobial resistance before and after PCV introduction: bacteraemia

Pre-PCV, using data averaged across 2001–05, the rate of non-susceptibility to penicillin (MIC > 0.06 mg/L) in bacteraemia was 6.5% while rates of resistance to erythromycin and tetracycline were 14.9% and 3.2%, respectively (Table 2). When compared to the most recent period, 2014–19, (i) there was no change in the prevalence of non-susceptibility to penicillin (6.2%), whereas rates of resistance to erythromycin decreased (5.9%) and tetracycline increased (7.4%) (Table 2); (ii) clindamycin resistance among erythromycin-resistant isolates, which was almost always constitutively expressed, increased from 12% to 78% (data not shown); and (iii) triple resistance averaged 2% with no clear trend (Table 2).

Isolates with penicillin MICs > 0.06 mg/L and resistance to both erythromycin and tetracycline were not sufficiently frequent to robustly compare pre- and post-PCV. The highest rates of triple resistance across all 19 years were in serotypes 15A (23%) and 6B (14%). While frequently resistant to erythromycin (58%), serotype 14 isolates typically lacked clindamycin cross resistance (data not shown). Serotype 33F was commonly resistant to erythromycin (21%) and tetracycline (15%) but remained susceptible to penicillin. In the case of serotype 3, resistance to tetracycline increased from 3% in 2001–05 to 21% in 2014–19, but remained low for erythromycin and penicillin (Table 2). Despite becoming the most prevalent serotype in bacteraemia, serotype 8 was rarely resistant to any agent (Table 2).

In 2001–05, serotype 14 represented 62% of resistance to erythromycin, with 9V, 23F and 6B collectively contributing around 17% (Table 3). Serotype 9V accounted for 53% of non-susceptibility to penicillin with 14, 23F and 6B adding another 30%, and serotypes 6B, 23F, 19A and 9V collectively accounted for 57% of the 37 isolates resistant to tetracycline. By 2014–19, serotypes 15A, 33F, 19A and 12F accounted for 66% of resistance to erythromycin, with considerable cross-resistance to clindamycin (data not shown); serotypes 3, 15A, 12F and 33F collectively represented 61% of resistance to tetracycline, while 58% of penicillin non-susceptibility was represented by serotypes 15A, 23B and 19A. The small burden of triple resistance was concentrated in serotypes 23F and 6B (8/12 isolates) in 2001–05 and in 15A and 19A (20/35 isolates) in 2014–19 (Table 3).

### Serotypes associated with antimicrobial resistance before and after PCV introduction: CA-LRTI

In 2005/06—before PCV7's introduction—6.8% of CA-LRTI pneumococci isolates were I or R to penicillin (MIC > 0.06 mg/L), whereas 11.6% and 8.4% were resistant to erythromycin and tetracycline, respectively (Table 4). Averaged from 2013/14 to 2018/19 (i.e. from three years after introduction of PCV13), these rates were higher, at 13.7%, 17.4% and 15.3% for penicillin, erythromycin and tetracycline, respectively (Table 4); moreover, clindamycin resistance among erythromycin-resistant isolates increased from 44% to 76% (data not shown). Triply resistant isolates (i.e. erythromycin-resistant, tetracycline-resistant, penicillin-non-susceptible) increased from 3% to 7%.

Considering all CA-LRTI isolates with serotype data, the highest rates of triple resistance were in serotypes 15A (35%), 19A (25%), 6B (22%), 14 (21%) and 19F (19%). As in bacteraemia, CA-LRTI isolates of serotype 3 showed a notable increase in resistance to tetracycline (only), from none of 51 isolates in 2005/06 to 46/172 (27%) in 2013/14–2018/19; serotype 23B (*N* = 121 in these 6 years) was commonly non-susceptible to penicillin (29%), but less frequently resistant to erythromycin (10%) or tetracycline (2%). Resistance/non-susceptibility rates for the 84 serotype 8 isolates from CA-LRTI (tetracycline 10%, erythromycin 6%, penicillin 4%) (Table 4) were distinctly higher than for bacteraemia isolates (rates 1%, 0% and 0%, respectively) in the last six surveillance years (Table 2).

In 2005/06, serotypes 14, 19F and 6B and serogroup 15 accounted for 69% of 87 erythromycin-resistant isolates; serotypes 14, 9V, 19F and 6B accounted for 75% of 51 isolates non-susceptible to penicillin and serotypes 6B, 14 and 19F for 52% of the 63 with tetracycline resistance (Table 5). Across the final six seasons, from 2013/14, serotypes 15A, 19F, 19A and 33F collectively accounted for 52% of resistance to erythromycin (and 65% of that to clindamycin) (data not shown); serotypes 15A, 23B, 35B and 19F accounted for 58% of penicillin non-susceptibility; serotypes 15A, 19F and 3 represented 53% of resistance to tetracycline (Table 5). Serotypes 14, 6B and 19F together accounted for three-quarters of all triple resistance in 2005/06, as did 15A, 19F and 19A in 2013/14–2018/19 (Table 5).

### Direct and indirect impact of PCV vaccination on serotype prevalence

We compared serotype distributions across two periods (2001–05 versus 2014–19 for bacteraemia and 2005/06 versus 2013/14–2018/19 for CA-LRTI) in two age categories: those aged ≤9 years (‘children’) and those aged ≥16 years (‘adults’) (Table 6). Given that PCV7 deployment in 2006 included a catch-up programme to reach children born in or after September 2004, it is unlikely that anyone aged ≥16 years when the surveillance ended in 2019 would have received a conjugate vaccine routinely, whereas all those aged ≤9 years in the latter periods should have been offered vaccination, with most accepting.

In the period from 2001 to 2005, 76% of bacteraemia isolates from children and 46% of those from adults belonged to PCV7 types (Table 6). On the other hand, PCV13-non7 serotypes were more prevalent from adults than children (24% versus 14%), as were PPV23-nonPCV serotypes (23% adults versus 5% children). Non-vaccine serotypes were rare in both children and adults (6% and 7%, respectively). By the more recent (2014–19) period, the proportion of PCV7 vaccine serotypes in bacteraemia had decreased to 6% for children and 3% for adults. The proportion of bacteraemia PCV13-non7 serotype isolates in children remained similar to that in 2001–05 while there were large increases in the proportion of PPV23-nonPCV isolates (from 5% to 38%) and non-vaccine types (from 6% to 39%). Among adults, the proportion of PCV13-non7 serotypes decreased slightly, with falls in serotypes 1 and 6A partially offset by rises in other serotypes; in addition, there was a major rise of PPV23-nonPCV serotypes (especially) and non-vaccine types. Of note, and likely reflecting the success of vaccination, the proportion of bacteraemia *S. pneumoniae* isolates from children halved between 2001–2005 (126/1149, 11%) and 2014–2019 (80/1336, 6%).

In the case of CA-LRTI, the proportion of isolates with serotypes belonging to the PCV7 group was similar for children (≤9 years; 44%) and adults (≥16 years; 42%) in 2005/06 (Table 6). By 2013/14–2018/19, these proportions had decreased 10-fold, to 4% to 5%, for both children and adults. By contrast, the proportion of PCV13-non7 isolates was little changed for either demographic. Balancing the falls in PCV7 serotypes, there were large increases in the proportions of isolates with PPV23-nonPCV and non-vaccine serotypes for both adults and children. The rise in the proportion of PPV23-nonPCV serotypes, from 3% to 31% for children and 9% to 32% for adults, was particularly striking. The proportion of CA-LRTI *S. pneumoniae* isolates from children ≤ 9 years almost halved between 2001/02–2005/06 (360/5078, 7%) and 2013/14–2018/19 (78/2180, 4%), again likely reflecting vaccine-mediated protection; in the same periods, the proportion of isolates that were from patients aged ≥65 years increased from 44% (2212/5078) to 51% (1104/2180).

### Impact of PPV23 vaccination on serotype prevalence

PPV23 serotypes, many later included in PCV formulations, accounted for close to 90% of bacteraemia pneumococci each year from 2001 to 2008, i.e. including five full years after its introduction in August 2003 (initially for those aged  ≥ 80, expanded to those aged  ≥ 65 in April 2005), indicating little or no immediate epidemiological effect (Figure 1). More recently, several PPV23-nonPCV serotypes, e.g. 8, 9N 12F and 22F, proliferated to become predominant bacteraemia types (Figure 3c).

To further investigate the impact of PPV23, we analysed, by age group, the proportion of PPV23-nonPCV serotypes among all non-PCV13 isolates before and after PCV deployment (Table 7). For bacteraemia among those aged 0–64 years, who are unlikely to have received PPV23, the proportions of PPV23-nonPCV13 isolates among all non-PCV13 serotypes remained similar both pre-PCV (76.5%) and post-PCV (75.1%), whereas this proportion decreased (73.8% pre-PCV, 62.9% post-PCV) among those aged ≥65 years, most of whom will have been vaccinated with PPV23. In contrast, the proportion of PPV23-nonPCV13 serotypes among CA-LRTI pneumococci increased similarly in both age groups post-PCV, with no strong evidence of aged-related difference.

## Discussion

Collection and centralized testing of *S. pneumoniae* isolates from the UK and Ireland offer insight into serotype trends for both invasive and respiratory disease during the first two decades of the 21st century, including after introduction of PCV vaccination for children and PPV23 for older adults.

Changes in serotype distribution for bacteraemia mirror global trends, which show PCV serotypes declining wherever childhood PCV is introduced.^7,17,18^ A reduction of PCV7/13 serotypes causing CA-LRTI is consistent with previous reports for England^10,19^ and internationally.^9,20^ The decrease in PCV serotypes was more striking in bacteraemia than CA-LRTI, reflecting the fact that the most strongly displaced serotypes—1 and 14—initially were more prominent in bacteraemia. After replacement of PCV7 by PCV13, three of the six PCV13-non7 serotypes—5, 6A and 7F—remained at, or were reduced to, very low levels in both bacteraemia and CA-LRTI; however, serotype 19A persisted and serotype 3 expanded after deployment of PCV13.

The persistence and increase of serotype 3, despite inclusion in PCV13, are reported by others internationally, particularly across Europe.^9,21,22^ Several factors may be involved. First, pre-PCV7, serotype 3 IPD was uncommon in young children but more prevalent in older children and adults.^23^ Accordingly, ‘success’, as measured by relative serotype prevalence, would require a particularly strong herd effect, which clearly was not obtained. Secondly, PCV13 is less immunogenic for serotype 3 than other serotypes included in the vaccine,^24^ with briefer protection and lower effectiveness.^25^ Thirdly, expansion of a particular lineage (Clade II), which has a changed antigenic profile and tetracycline resistance, may be contributing to the success of this serotype,^26^ especially given advocacy of doxycycline for community respiratory infections.^27^ Notably, we observed an increase in the percentage of tetracycline resistance in serotype 3 among both bacteraemia and CA-LRTI isolates. Lastly, the physical attributes of the capsule of serotype 3 isolates may be important. These organisms have a thicker and more mucoid polysaccharide, which may hinder antibody-mediated opsonization and clearance; moreover, particles are shed, acting as decoy antibodies.^28^

Although not on the scale of serotype 3, the persistence of serotypes 19A (PCV13) and 19F (PCV7) (Figure 1a/b), also noted by others in the UK and continental Europe,^9,21,29^ is of concern. These serotypes are associated with antimicrobial resistance, often to multiple antimicrobials, and this may contribute to their success. Other likely contributory factors include some combination of fitness,^30^ lower vaccine effectiveness and accelerated waning of vaccine protection.^25^

Various non-PCV and non-vaccine types have partly replaced the displaced PCV7/13 types. Serotype 8, a PPV23-nonPCV serotype, became the most prevalent serotype in bacteraemia from 2014, as reported also by other UK and European surveillance.^19,21^ Curiously, serotype 8 has not become persistently prominent in CA-LRTI, implying either that it has an unusually high pneumonia to bacteraemia conversion rate or that many serotype 8 bacteraemias do not originate from pneumonia. Other expanding types include the PPV23-nonPCV serotypes 9N, 11A, 12F, 22F and 33F, along with the non-vaccine serotypes, 15A, 23A and 23B, as reported by others.^29,31,32^

Contrary to recent reports of an increased proportion of IPD associated with serotype 4 (PCV7) in 18–64-year-old adults in the UK and Spain,^33,34^ we did not identify persistence or expansion of this serotype. This re-emergence and increase of serotype 4—which may largely post-date the end of the BSAC Project—possibly reflect a recent capsular-switch event between serotype 4/ST10172 and serotype 12F/ST220.^35^ It requires close monitoring, as both serotypes 4 and 12F have been associated with multiple drug resistance.^36^

These BSAC data measure the relative prevalence of different serotypes. UKHSA data, which also measure case numbers, and therefore disease incidence, indicate a reduction in IPD incidence after deployment of PCV7 and 13 followed by a rebound among adults—who were protected by a herd effect—though not in children, who have received direct PCV7/13 vaccination.^10^ It is unclear whether these differences in trajectory are because the now-proliferating serotypes are less able to cause severe disease in children, or because direct PCV vaccination offers wider protection, beyond the included serotypes. Notably, and underscoring the vaccines’ direct efficacy, the proportion of BSAC isolates from children aged ≤9 years versus adults approximately halved from the pre-vaccine era for both bacteraemia and CA-LRTI (Table 6). For completeness, and while it occurred after the BSAC surveillance period, it is worth noting the suppression of IPD during the COVID-19 pandemic, followed by a subsequent rebound of the previously circulating serotypes.^33,37,38^

The small numbers of isolates of individual serotypes and the risk of confounding limited our ability to investigate resistance trends within serotypes and vaccine groups. Instead, we reviewed which serotypes were the main contributors to resistance in specified pre- (2001–05) and post-PCV deployment (2014–19) periods for three key antimicrobials that were tested in all years and both programmes. Pre-PCV, the dominant types associated with resistance were PCV7 or 13 serotypes, namely, 6B, 9V, 14, 19A, 19F and 23F. Pre-PCV, these accounted for much of the global problem with multi-drug resistant *S. pneumoniae*—an issue that was more acute in e.g. southern Europe, the US and South Africa than in the UK.^39^ Of these types, only 19A and 19F persisted (contributing up to 14% of resistance) in the later years of the BSAC Surveillance, long after deployment of PCV7 and 13, whereas types 6B, 9V, 14 and 23F had been displaced and contributed minimally. Serotype 15A—which became increasingly prominent after PCV deployment—is not covered by any current vaccine and is often associated with multiple resistance. We previously reported rise of serotype 15A,^40^ which also occurred internationally, including in Canada and the US, Germany and Japan^41,42^ For unknown reasons, this rise in the UK stalled around 2015. Rates of resistance were higher among respiratory than bacteraemia pneumococci, as reported previously.^43^

Whereas both the individual protective and epidemiological effects of PCVs are widely agreed, the effectiveness of PPV23 against vaccine-type IPD remains under debate.^44^ Protection estimates vary from 28% to 54% for adults aged 65–79 years, reducing with age, comorbidities and the elapsed time after vaccination.^45–47^ The present data show that (i) most now-predominant bacteraemia serotypes, in particular 3, 8, 9N, 12F, 19A and 22F, are represented in PPV23; (ii) these serotypes have increased in prevalence while PCV23 was in use; and (iii) PPV23 serotypes accounted for over 90% of all bacteraemia pneumococci throughout the period 2001–08, despite PPV entering wide use in older adults in 2003, with this proportion falling only once the herd effect of PCV7 became manifest (Figure 2a). All these points argue against substantial efficacy. Nevertheless, a further analysis does suggest a modest effect (Table 7): among those aged ≤64 years (unlikely to have received PCV23), the proportion of PCV23-non-PCV13 serotypes among all non-PCV13 serotypes was around 75%–77% in both 2001–05 and 2014–19, whereas, for the over 65s (likely to have received PPV23), it diminished from 74% to 63%. This is consistent with reduced expansion of PPV23-nonPCV in the population most likely to have been vaccinated. Caveats are that we do not know the vaccination status of the source patients, the severity of their illness, nor whether vulnerability to particular serotypes varies with age. For CA-LRTI, there was no suggestion of efficacy.

Wider limitations of the BSAC Surveillance Project are discussed elsewhere;^14^ however, challenges specific to these serotype data need mention. First, we measured relative serotype prevalence, not absolute prevalence, let alone incidence. Case numbers can fall even as the relative importance of a serotype increases; all that is required is for other serotypes to fall faster. Secondly, shifts in the serotype distribution may occur naturally irrespective of vaccination: serotype 14 was already decreasing in bacteraemia before introduction of PCV7 (Figure 3a); likewise, the rise of serotype 15A stalled despite antibiotic resistance and the lack of vaccine-mediated protection. Thirdly, whereas we have continuous data for bacteraemia isolates, we collected only a 1-year (2005/06) snapshot of pre-PCV CA-LRTI serotypes, with continuous data only beginning 3 years after deployment of PCV13. Lastly, the Project did not include genotyping, which would have informed changes in clonal lineages over time. Genotype is more fundamental than serotype because the same genotype can acquire different serotype determinants, as with multi-resistant ST63 expressing either serotype 8 or 15A capsular antigens.^41,48^

Vaccines have an important role in addressing the global challenge of antimicrobial resistance. By preventing bacterial and viral infections, vaccines can impact inappropriate and unnecessary prescribing of antibiotics, reducing the selective pressure for resistance.^49^ IPD continues to cause a high disease burden despite 21 years of PPV23 vaccination in older adults and vulnerable groups and 18 years of PCV vaccination in children. It is imperative to continue striving to reduce the incidence of IPD, non-bacteraemia pneumonia and more common infections, such as otitis media. In context, in 2023 Pfizer licensed PCV20 (PCV13 serotypes plus seven others, Table 1) and in the same year, the UK Joint Committee of Vaccination and Immunization recommended PCV20 as an alternative to PPV23 for those aged over 65 years and other vulnerable groups,^50^ but deployment has not yet commenced. MSD, meanwhile, have developed V116, a conjugate vaccine aiming to cover 21 serotypes currently prevalent in adults (Table 1).^51^ Further vaccines are in development, including a 24-valent PCV for adults from Vaxcyte.^52^ It remains to be seen which of these will be preferred. National vaccination committees will need to weigh the risk of resurgent serotype 15A versus resurgent 19F (both associated with multidrug resistance) and decide whether it is better to use the same or different vaccines in adults and children.

In conclusion, the data presented here corroborate bacteraemia serotype distribution trends reported by the UKHSA and others internationally and offer a comparison of serotypes among respiratory disease. Global surveillance of circulating serotypes and associated antimicrobial resistance remains a vital tool to inform pneumococcal vaccination and treatment policies to protect those most vulnerable to pneumococcal disease.

## Supplementary Material

dkaf253_Supplementary_Data

## Transparency declarations

S.M., A.C. and R.A. are employees of the UKHSA and work for the Antimicrobial Resistance and Healthcare Associated Infections Reference Unit (AMRHAI), which has received financial support for conference attendance, research projects or contracted evaluations from numerous sources, including Accelerate Diagnostics, Allecra Therapeutics, AstraZeneca UK Ltd, AusDiagnostics, Basilea Pharmaceutica, Becton Dickinson Diagnostics, bioMérieux, Bio-Rad Laboratories, BSAC, Cepheid, Check-Points B.V., UK Department of Health, Enigma Diagnostics, Food Standards Agency, GlaxoSmithKline Services Ltd, Helperby Therapeutics, Henry Stewart Talks, IHMA Ltd, Innovate UK, Integra holdings, MSD, Meiji Seika Pharma Co. Ltd, Mobidiag, Momentum Biosciences Ltd, Neem Biotech, Nordic Pharma Ltd, Norgine Pharmaceuticals, Paratek Pharmaceuticals, Roche, Smith & Nephew UK Ltd, Shionogi & Co. Ltd, Trius Therapeutics, VenatoRx Pharmaceuticals and Wockhardt Ltd. C.H. was employed by Una Health Limited and QuantuMDx Group Limited during the period of data analysis and manuscript preparation. M.A. is a Trustee of the BSAC Council and is employed by MSD (UK) Limited, London, UK. M.A. has shareholdings in Merck & Co., Inc., Rahway, NJ, USA. C.L. is a Trustee of the BSAC Council and is employed by Shionogi B.V. B.J.P.: consultancy: Genpax. D.M.L.: advisory boards or *ad hoc* consultancy: AdjuTec, AstraZeneca, Centauri, GenPax, GSK, Lipovation, Meiji, Paion, Shionogi, Sumitovant, Thermo Fisher and Wockhardt; paid lectures: bioMérieux, Pfizer, Shionogi and Zuellig Pharma; and relevant shareholdings or options: GenPax, GSK, Merck & Co., Inc., and Revvity amounting to less than 10% of portfolio value. D.M.L. also has shareholdings in Arecor, Genincode, Probiotix, Rua Life Sciences, SkinBiotherapeutics and VericiDx (all with research/products pertinent to medicines or diagnostics) through Enterprise Investment Schemes, but has no authority to trade these holdings directly. *All other authors* have nothing to declare.

## Supplementary data

Tables S1 and S2 are available as Supplementary data at *JAC*  Online.
