# Supplementary material for: Trends of serotypes and resistance among Streptococcus pneumoniae in the UK and Ireland (1999–2019)
Source: J Antimicrob Chemother. 2025 Oct 27;80(Suppl 4):iv72–86. doi: 10.1093/jac/dkaf253 (PMC12556646; doi:10.1093/jac/dkaf253)
Supplement: dkaf253_Supplementary_Data [file dkaf253_supplementary_data.pdf]

Trends of serotypes and resistance among *Streptococcus pneumoniae* in the UK and Ireland (1999–2019)

SUPPLEMENTARY INFORMATION

Three companion papers provide extensive further information to complement the focus on pneumococcal serotype distribution and evolution in this paper. Allen *et al*. detail the full methods of the BSAC Resistance Surveillance Project,[1] while Reynolds *et al*. document the two collections of *S. pneumoniae*, from patients with bacteraemia and community-associated lower respiratory tract infection (CA-LRTI) and analyse their resistance to a wide range of antimicrobial agents.[2-3]

Contents

Collection of *S. pneumoniae* isolates 1

Central testing laboratories 1

Table S1. Isolate collection periods, quotas and targets 1

Table S2. Actual numbers of *S. pneumoniae* isolates tested, and centres contributing by year 2

References 2

Collection of *S. pneumoniae* isolates

Central testing laboratories

Central testing for CA-LRTI isolates was at GR Micro, London (later Quotient Bioresearch, then LGC, Fordham, UK) in 1999/2000–2012/13 and at the Antimicrobial Resistance and Healthcare-Associated Infections Reference Unit (AMRHAI) of Public Health England (later the UK Health Security Agency), Colindale, London, in 2013/14–2018/19.

Central testing for bacteraemia isolates was at AMRHAI throughout.

Table S1. Isolate collection periods, quotas and targets

Annual collection	Collection period	Target N of centres	*S. pneumoniae* isolates Quota per lab	Target: total
**Bacteraemia**				
2001–2007	1 January – 31 December	25	10	250
2008–2009	1 January – 31 December	25	10	250
2010–2015	1 January – 31 December	40	7	280
2016–2019	1 January – 31 December	25	10	250
**CA-LRTI**				
1999/00–2007/08	1 October – 30 April	20	50	1000
2008/09–2009/10	1 October – 30 September	20	25	500
2010/11–2014/15	1 October – 30 September	40	14	560
2015/16–2018/19	1 October – 30 September	25	20	500

Table S2. Actual numbers of *S. pneumoniae* isolates tested, and centres contributing by year

	**Bacteraemia**			**CA-LRTI**	
Year	N of centres	N of isolates	Season	N of centres	N of isolates
			1999/2000	20	661
2001	24	227	2000/01	20	667
2002	25	220	2001/02	22	699
2003	25	239	2002/03	22	772
2004	25	241	2003/04	22	785
2005	25	230	2004/05	21	750
2006	25	231	2005/06	22	749
2007	24	216	2006/07	21	727
2008	23	201	2007/08	23	809
2009	25	211	2008/09	22	451
2010	39	249	2009/10	23	480
2011	36	230	2010/11	38	420
2012	38	229	2011/12	37	383
2013	37	235	2012/13	32	345
2014	39	247	2013/14	36	375
2015	40	244	2014/15	37	429
2016	24	220	2015/16	24	358
2017	23	208	2016/17	24	345
2018	23	208	2017/18	23	325
2019	24	215	2018/19	24	351
Total	(79)	4301	Total	(77)	10,881

References

[1] Allen M, Reynolds R, Mushtaq S *et al.* The British Society for Antimicrobial Chemotherapy Resistance Surveillance Project: methods and limitations. *J Antimicrob Chemother* 2025; **80** (Suppl 4): iv7–iv21.

[2] Reynolds R, Mushtaq S, Hope R *et al.* Antimicrobial resistance among Gram-positive agents of bacteraemia in the UK and Ireland: trends from 2001 to 2019. *J Antimicrob Chemother* 2025; **80** (Suppl 4): iv22–iv35.

[3] Reynolds R, Felmingham D, Mushtaq S *et al.* Antimicrobial resistance among agents of community-associated lower respiratory tract infection in the UK and Ireland: trends from 1999/2000 to 2018/2019. *J Antimicrob Chemother* 2025; **80** (Suppl 4): iv60–iv71.
